# Genomic data on breast cancer transcript profile modulation by 17beta-hydroxysteroid dehydrogenase type 1 and 17-beta-estradiol

**DOI:** 10.1016/j.dib.2016.11.010

**Published:** 2016-11-09

**Authors:** Juliette A. Aka, Ezequiel-Luis Calvo, Sheng-Xiang Lin

**Affiliations:** Laboratory of Molecular Endocrinology and Oncology, Centre Hospitalier Universitaire de Québec Research Centre (CHUQ, CHUL) and Department of Molecular Medicine, Laval University, 2705 boulevard Laurier, Québec G1V 4G2, Canada

## Abstract

The data presented here are related to the research article entitled “Estradiol-independent modulation of breast cancer transcript profile by 17beta-hydroxysteroid dehydrogenase type 1” (J.A. Aka, E.L. Calvo, S.X. Lin, 2016) [1]. We evaluated the effect of the steroidal enzyme 17β-HSD1 and its product, the estrogenic hormone 17-beta-estradiol (E2), on gene transcription profile of breast cancer cells. RNA interference technique was used to knock down the 17β-HSD1 gene (*HSD17B1*) in the hormone-dependent breast cancer cell line T47D in steroid-deprived medium. Transfected cells were subsequently treated with E2, and microarray analyses (with three contrasts) were used to investigate (i) the effect of 17β-HSD1 expression on breast cancer cell transcript profile in steroid-deprived condition, (ii) the effect of E2 on breast cancer gene expression and (iii) if E2 affects gene regulation by 17β-HSD1. Functional enrichments of the differentially expressed genes were assessed using Ingenuity Pathway Analysis (IPA). Here, we showed data on 140 genes that are induced or repressed 1.5 time or higher (*p* < 0.05) in the *HSD17B1-*silenced and E2-treated T47D cells revealed by microarray analysis, and presented the 14 functional terms found in the cancer and in the cell death and survival categories revealed by the IPA biological function analysis. Data on IPA Canonical Pathway and network analyses is also presented. Further discussion on gene regulation by 17β-HSD1 and E2 is provided in the accompanying publication [1].

**Specifications Table**

**Table:** 

Subject area	Biology
More specific subject area	Breast cancer
Type of data	Table, Figure
How data was acquired	Microarray analysis: microarray was processed using Affymetrix GeneChip Whole Transcript (WT) Sense Target Labeling Assay and quantified Affymetrix image files were analyzed using the *Bioconductor* package OneChannelGUI into the statistical software environment R.
	Bioinformatics analysis: functional enrichment analysis was done using the gene list from the microarray analysis and Ingenuity pathway core analysis (IPA^®^, QIAGEN Redwood City).
Data format	Analyzed and filtered data
Experimental factors	T47D cells were transfected with 17β-HSD1 siRNAs followed by estradiol treatment two days later for an additional two days, and total RNA was extracted for analysis.
Experimental features	3×10^5^ T47D cells were transfected in 6-well plates in charcoal-treated medium with 200 nM mixed 17β-HSD1 specific siRNAs or with negative control siRNA. Two days later, transfected cells were treated with 1 nM estradiol or *ethanol* as a *vehicle control* in fresh charcoal-treated medium and cells were incubated for two additional days before RNA extraction and analysis.
Data source location	N/A
Data accessibility	Data is available within this article and available at the NCBI database via Gene Expression Omnibus (GEO accession number GSE77345).

**Value of the data**

• Provide information on genes regulated by 17β-HSD1 and its product estradiol, useful for further studies on breast cancer cell mechanisms.
• Can contribute to elucidate hormone-independent cell growth pathways in hormone-dependent breast cancer cells.
• May stimulate further research on understanding 17β-HSD1 roles in breast cancer development.

## 1 Data

**Fig. 1:**
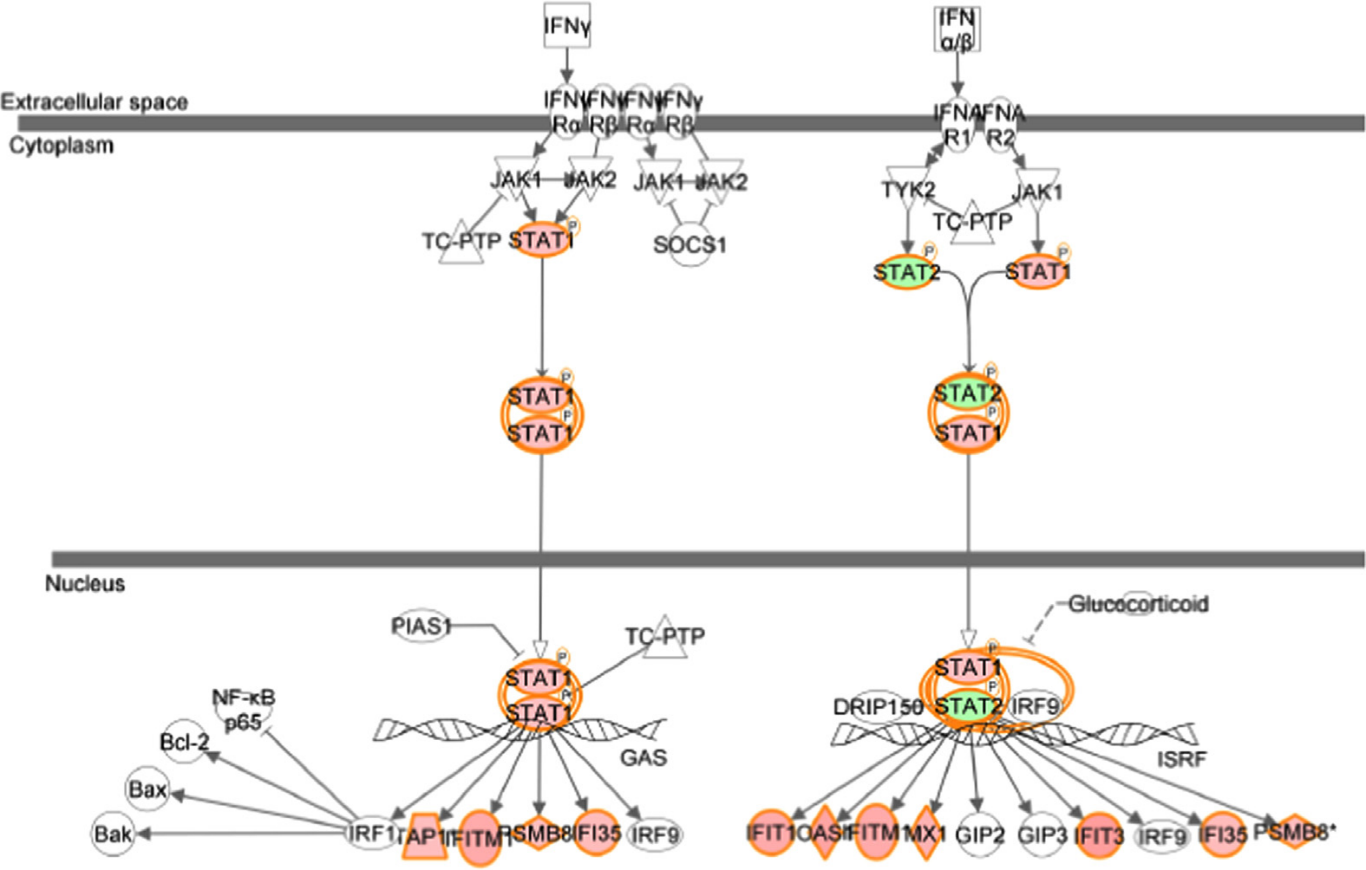
IPA Canonical Pathway analysis showing the interferon signaling pathway across the NC *vs.* NC+E2 contrast data.

**Fig. 2:**
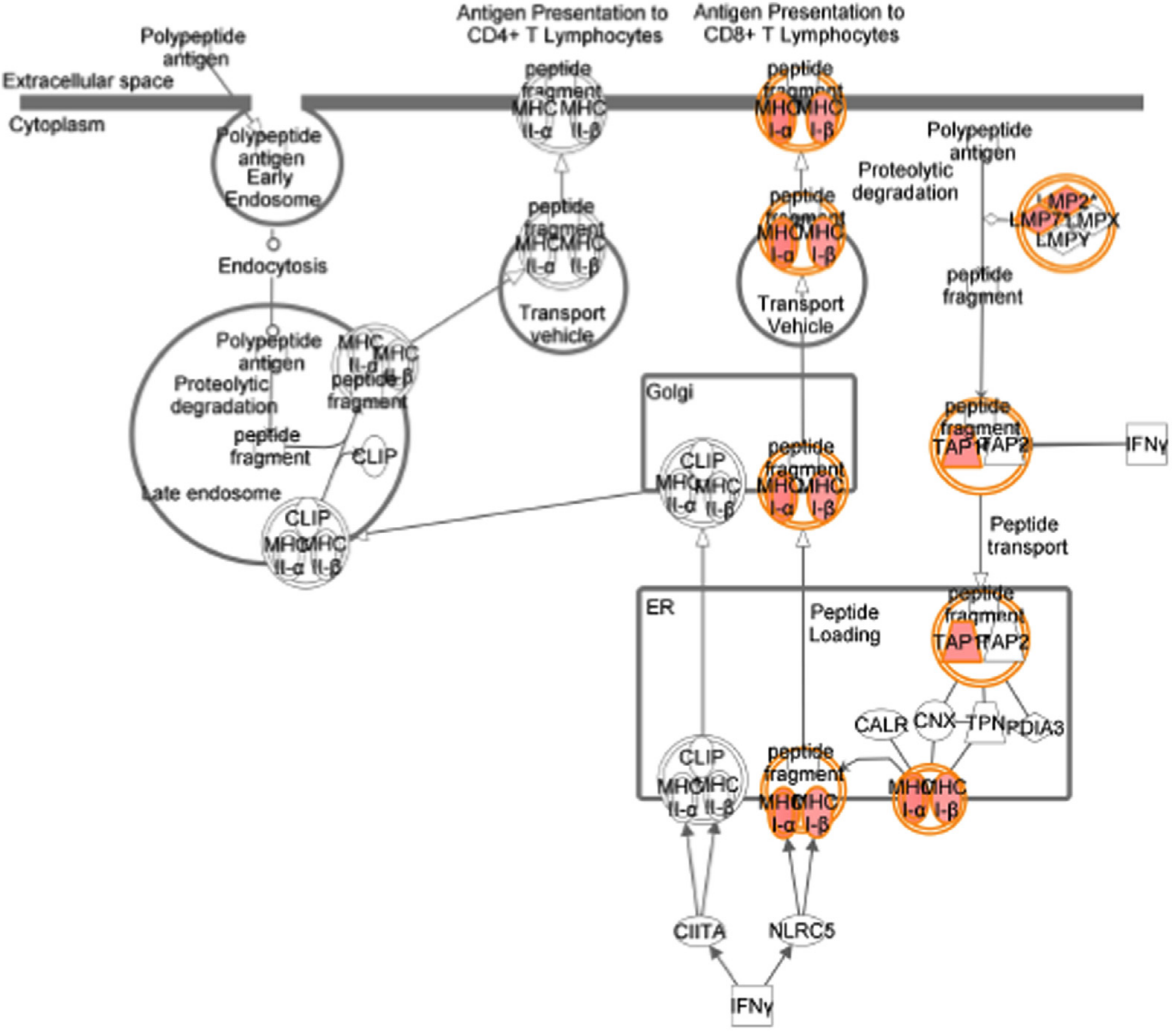
Canonical pathways by IPA: antigen presentation pathway in the NC *vs.* si17B1 contrast.

**Fig. 3:**
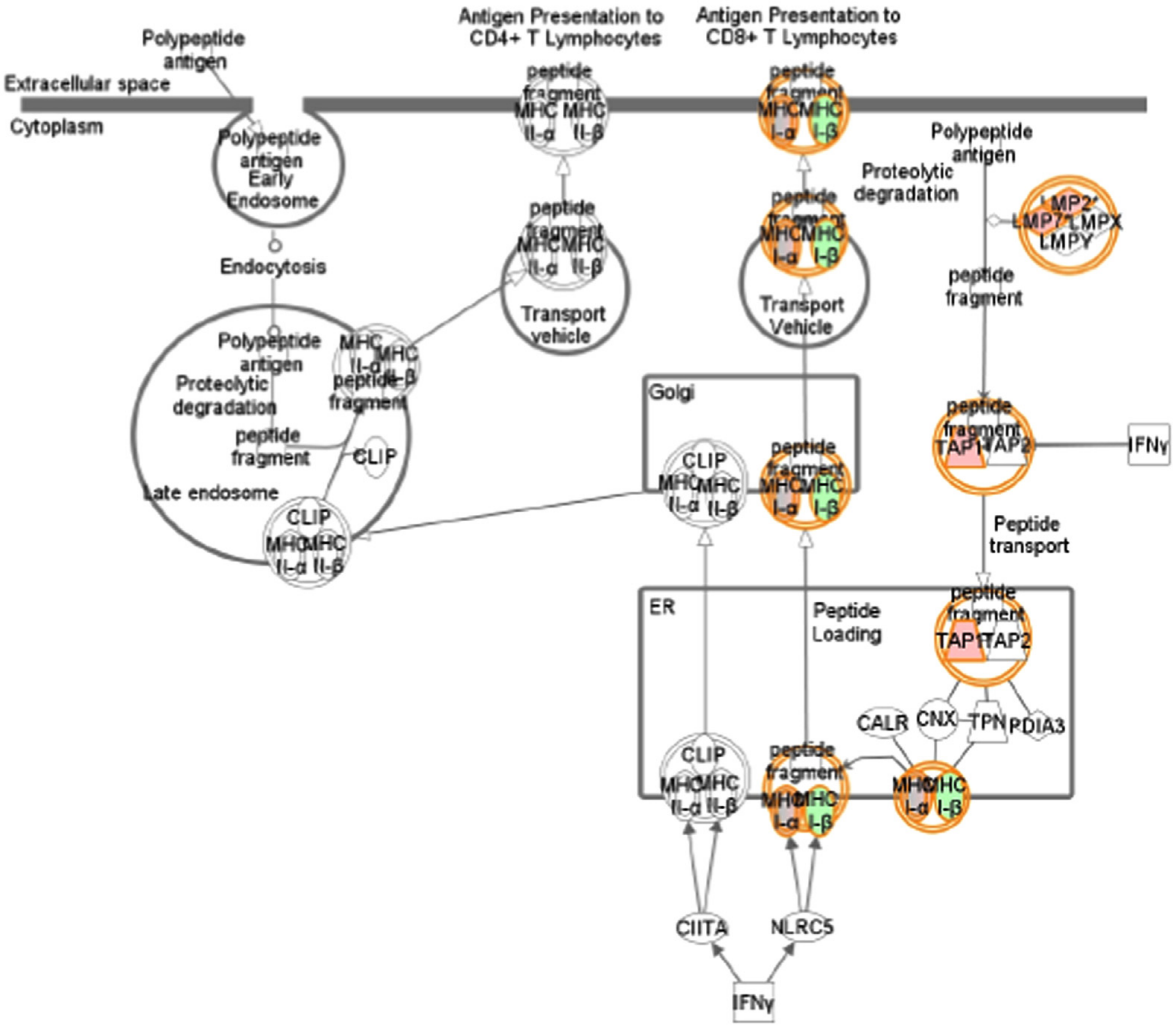
Canonical pathways by IPA: antigen presentation pathway in the NC *vs.* NC+E2 contrast.

**Fig. 4:**
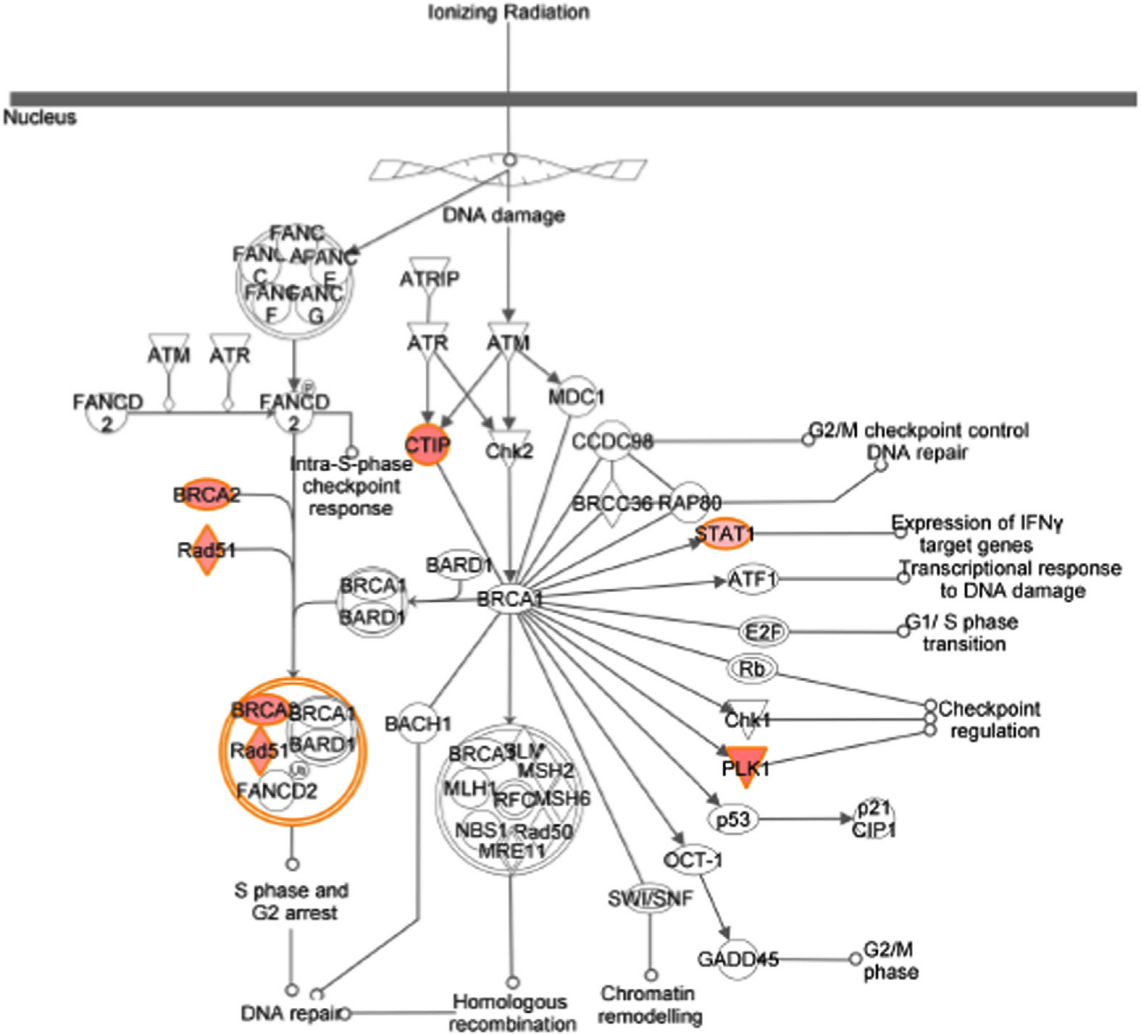
Canonical pathways by IPA: role of BRCA1 in DNA damage response in the NC *vs.* NC+E2 contrast.

Table 1 showed data on gene expression profile in T47D cells (genes regulated 1.5 time or higher) transfected with 17β-HSD1 siRNAs (si17B1) or negative control siRNA (NC), and treated with 1 nM estradiol (E2), revealed by microarray analysis with contrast NC+E2 *vs.* si17B1+E2 (see Table 5 for additional information). Table 2 showed the 14 functional terms found in the cancer category of the IPA biological function analysis of 208 genes from three fold change lists (genes which fold change equal or higher than 1.5 in at least one contrast), generated by the three contrasts (NC *vs.* si17B1; NC *vs.* NC+E2; and NC+E2 *vs.* si17B1+E2) of microarray analyses (see Table 5 for contrast description). Table 3 showed the 14 functional terms found in the cell death and survival category of the IPA biological function analysis of 208 genes generated by the three contrasts of microarray analyses. Table 4 showed data on the IPA network analysis of 208 genes from the three contrasts (NC *vs.* si17B1; NC *vs.* NC+E2; and NC+E2 *vs.* si17B1+E2) of the microarray analysis. Fig. 1, Fig. 2, Fig. 3, Fig. 4 showed IPA Canonical pathway analyses for interferon signaling pathway in the NC *vs.* NC+E2 contrast (Fig. 1), antigen presentation pathway in the NC *vs.* si17B1 contrast (Fig. 2), antigen presentation pathway in the NC *vs.* NC+E2 contrast (Fig. 3), and role of BRCA1 in DNA damage response in the NC *vs.* NC+E2 contrast (Fig. 4).

## 2 Experimental design, materials and methods

### 2.1 Cell culture, siRNA transfections, steroid treatment and RNA preparation

T47D cells were obtained from the American Type Culture Collection (ATCC) and were cultured as described in ref [1]. The detailed procedure of siRNA transfections, steroid treatment and RNA preparation have been described in ref [1]. Briefly, two days before transfection, T47D cells were cultured in dextran-coated charcoal-treated medium; on the transfection day, 3×10^5^ cells were reverse-transfected in 6-well plates with 200 nM mixed 17β-HSD1 specific siRNAs [2], [3] (si17B1) or with Scramble siRNA used as negative control siRNA (NC) using Lipofectamine siRNAMax (Invitrogen), and cells were incubated in steroid deprived medium. Two days after transfection, cell culture media were replaced by fresh charcoal-treated medium containing either the steroid estradiol (1 nM) or *ethanol* as a *vehicle control* (*see* Table 5), and cells were incubated for two more days before RNA extraction using Trizol Reagent (Invitogen). The RNA samples included two independent biological replicates, coming from two independent cell culture experiments, for a total of eight RNA samples.

### 2.2 Microarray processing

RNA samples were processed according to the manufacturer׳s recommended procedures on GeneChip Whole Transcript (WT) Sense Target Labeling Assay from Affymetrix (http://www.affymetrix.com/support/downloads/manuals/wt_sensetarget_label_manual.pdf). The assay was started with 0.2 µg of each T47D cells RNA samples and the protocol is based on the principle of performing one cycle of cDNA synthesis and in vitro transcription (IVT) for target amplification to generate cRNA following by reverse transcription reactions to synthesis the WT cDNA. About 2.7 µg sample of fragmented cDNAs was used to hybridize human oligonucleotide array Gene 1.0 ST (Genechip; Affymetrix). The array comprised more than 750,000 unique 25-mer oligonucleotides constituting over 28,000 gene-level probe sets of the human genome. The cDNA probe corresponding to each biological repetition for each condition was hybridized on separate arrays. After hybridization, chips were processed using the Affymetrix GeneChip Fluidic Station 450 (protocol F450_0007). Chips were scanned with a GeneChip scanner 3000 7G (Affymetrix) and images were extracted with the GeneChip operating software (Affymetrix GCOS v1.4). The microarray processing was performed at the DNA Biochip Platform service at CHU de Québec - CHUL Research Centre (Québec, Canada).

### 2.3 Microarray analysis

The microarray analysis has been described in the accompanying paper [1]. *Quantified Affymetrix image* files (".CEL" files) for each of the treatment conditions (including two independent replicates per treatment condition) were used to perform the microarray analyses using the *Bioconductor* package OneChannelGUI [4], [5] in the statistical software environment R. Three contrasts (see Table 5) were using the RMA method [6]. Data filtering was performed at signal feature level by interquantile range (IQR) then by intensity. To identify differentially expressed genes, gene expression intensity was compared using a moderated *t*-test and a Bayes smoothing approach developed for a low number of replicates [7], and the false discovery rate was estimated from *P-values* derived from the moderated *t*-test statistics for correction for the effect of multiple testing [8]. Genes were considered to be significantly differentially expressed if *p-values* were <0.05. The log_2_ transformed signal intensities were averaged, and the mean value was used to compute the fold changes. Genes that were differentially expressed 1.5-fold or higher were considered for subsequent analyses. Our microarray data is available in the Gene Expression Omnibus (GEO) repository, accession number GSE77345.

### 2.4 Functional enrichment analysis

Ingenuity pathway analysis (IPA^®^, QIAGEN Redwood City, www.qiagen.com/ingenuity) was used to assess the functional enrichment of the 208 modulated genes revealed by the three-contrast microarray analysis (genes which fold change equal or higher than 1.5 in at least one contrast). Three analyses made by IPA were presented here: identification of biological functions, gene networks and canonical pathways (see ref for additional information). Criteria used for the IPA analyses have been described in the accompanying research article [1].

## Figures and Tables

**Table 1:** List of the 140 genes induced or repressed 1.5 time or higher (*p*<0.05) in T47D cells after transfection with 17β-HSD1 siRNAs (si17B1) or negative control siRNA (NC) for two days and cell treatment with 1 nM estradiol (E2). Data was obtained from microarray analysis using contrast NC+E2 *vs.* si17B1+E2 (see Table 5).

Symbol	Description	Fold change
CDH10	Cadherin 10, type 2 (T2-cadherin)	5,2
IFI44	Interferon-induced protein 44	4,7
IFIT2	Interferon-induced protein with tetratricopeptide repeats 2	4,6
IFIT3	Interferon-induced protein with tetratricopeptide repeats 3	4,4
RSAD2	Radical S-adenosyl methionine domain containing 2	4,3
DDX60L	DEAD (Asp-Glu-Ala-Asp) box polypeptide 60-like	4,0
CCL5	Chemokine (C-C motif) ligand 5	3,4
IFIT1	Interferon-induced protein with tetratricopeptide repeats 1	3,4
ARL4D	ADP-ribosylation factor-like 4D	3,3
NAALADL2	N-acetylated alpha-linked acidic dipeptidase-like 2	3,2
BTN3A2	Butyrophilin, subfamily 3, member A2	3,1
LBA1	Lupus brain antigen 1	3,1
DDX60	DEAD (Asp-Glu-Ala-Asp) box polypeptide 60	3,0
XAF1	XIAP associated factor 1	2,9
MDGA2	MAM domain containing glycosylphosphatidylinositol anchor 2	2,9
OAS2	2׳-5׳-oligoadenylate synthetase 2, 69/71 kDa	2,9
RARRES3	Retinoic acid receptor responder (tazarotene induced)	2,9
HCP5	HLA complex P5	2,9
OASL	2׳-5׳-oligoadenylate synthetase-like	2,9
PARP14	Poly (ADP-ribose) polymerase family, member 14	2,8
IFITM3	Interferon induced transmembrane protein 3 (1-8U)	2,8
LAMP3	Lysosomal-associated membrane protein 3	2,7
AKAP6	A kinase (PRKA) anchor protein 6	2,7
PSMB9	Proteasome subunit beta type-9	2,7
BTN3A1	Butyrophilin, subfamily 3, member A1	2,7
RANBP3L	RAN binding protein 3-like	2,6
HLA-F	Major histocompatibility complex, class I, F	2,5
IFITM1	Interferon induced transmembrane protein 1 (9–27)	2,5
DDX58	DEAD (Asp–Glu–Ala–Asp) box polypeptide 58	2,5
BTN3A3	Butyrophilin, subfamily 3, member A3	2,5
ERBB4	V-erb-a erythroblastic leukemia viral oncogene homolog 4 (avian)	2,5
HLA-H	Major histocompatibility complex, class I, H (pseudogene)	2,4
HLA-A	Major histocompatibility complex, class I, A	2,4
PARP9	Poly (ADP-ribose) polymerase family, member 9	2,4
HLA-C	Major histocompatibility complex, class I, C	2,3
CTSO	Cathepsin O	2,3
L3MBTL3	L(3)mbt-like 3 (Drosophila)	2,3
HLA-E	Major histocompatibility complex, class I, E	2,3
IFI35	Interferon-induced protein 35	2,3
HLA-B	Major histocompatibility complex, class I, B	2,3
APOL1	Apolipoprotein L, 1	2,3
HLA-G	Major histocompatibility complex, class I, G	2,3
HERC6	Hect domain and RLD 6	2,2
HERC5	Hect domain and RLD 5	2,2
IFIH1	Interferon induced with helicase C domain 1	2,2
SLC46A3	Solute carrier family 46, member 3	2,2
VGLL1	Vestigial like 1 (Drosophila)	2,2
TAP1	Transporter 1, ATP-binding cassette, sub-family B (MDR/TAP)	2,2
FGF12	Fibroblast growth factor 12	2,2
PLSCR1	Phospholipid scramblase 1	2,2
HLA-A29,1	Major histocompatibility complex class I HLA-A29,1	2,2
LGALS3BP	Lectin, galactoside-binding, soluble, 3 binding protein	2,2
SAMD9	Sterile alpha motif domain containing 9	2,2
ATP1B1	Atpase, Na+/K+ transporting, beta 1 polypeptide	2,1
OAS1	2׳,5׳-oligoadenylate synthetase 1, 40/46kda	2,1
PSMB8	Proteasome subunit beta type-9	2,1
OAS3	2׳-5׳-oligoadenylate synthetase 3, 100kda	2,1
CENTD1	Centaurin, delta 1	2,1
IFI27	Interferon, alpha-inducible protein 27	2,1
DHX58	DEXH (Asp–Glu–X-His) box polypeptide 58	2,1
STAT2	Signal transducer and activator of transcription 2, 113kda	2,0
LAMC1	Laminin, gamma 1 (formerly LAMB2)	2,0
THSD7A	Thrombospondin, type I, domain containing 7A	2,0
TGFB2	Transforming growth factor, beta 2	2,0
USP18	Ubiquitin specific peptidase 18	2,0
MX1	Interferon-induced GTP-binding protein Mx1	2,0
B2M	Beta-2-microglobulin	2,0
DTX3L	Deltex 3-like (Drosophila)	2,0
ERAP1	Endoplasmic reticulum aminopeptidase 1	2,0
SLC15A3	Solute carrier family 15, member 3	1,9
CFB /// C2	Complement factor B /// complement component 2	1,9
TNFSF10	Tumor necrosis factor (ligand) superfamily, member 10	1,9
ROBO2	Roundabout homolog 2	1,9
IL1R1	Interleukin 1 receptor, type I	1,9
RAB27B	RAB27B, member RAS oncogene family	1,9
RTP4	Receptor (chemosensory) transporter protein 4	1,9
STAT1	Signal transducer and activator of transcription 1, 91kda	1,9
CASP4	Caspase 4, apoptosis-related cysteine peptidase	1,9
INSIG2	Insulin induced gene 2	1,9
DKFZP434B2016	Similar to hypothetical protein LOC284701	1,9
SMARCA1	Nucleosome-remodeling factor subunit SNF2L	1,8
LIPH	Lipase, member H	1,8
ZNFX1	Zinc finger, NFX1-type containing 1	1,8
UBP1	Upstream binding protein 1 (LBP-1a)	1,8
KLF8	Kruppel-like factor 8	1,8
LRRK2	Leucine-rich repeat kinase 2	1,8
LTBP1	Latent transforming growth factor beta binding protein 1	1,8
EVI1	Ecotropic viral integration site 1	1,8
FBXO32	F-box protein 32	1,7
BCAS1	Breast carcinoma amplified sequence 1	1,7
ALCAM	Activated leukocyte cell adhesion molecule	1,7
MTERFD3	MTERF domain containing 3	1,6
MMP16	Matrix metallopeptidase 16	1,6
LOC390345	Similar to ribosomal protein L10	1,6
CITED2	Cbp/p300-interacting transactivator with Glu/Asp-rich carboxy-terminal domain 2	1,6
CYP4Z2P	Cytochrome P450, family 4, subfamily Z, polypeptide 2 pseudogene	1,6
RNF43	Ring finger protein 43	1,6
ZNF175	Zinc finger protein 175	1,5
SEPP1	Selenoprotein P, plasma, 1	1,5
EPAS1	Endothelial PAS domain protein 1	1,5
FAM115A	Family with sequence similarity 115, member A	1,5
SEMA6A	Semaphorin 6A	1,5
NBEA	Neurobeachin	1,5
CSAD	Cysteine sulfinic acid decarboxylase	1,5
SNX24	Sorting nexin 24	−1,5
RBBP8	Retinoblastoma binding protein 8	−1,5
PTTG1	Pituitary tumor-transforming 1	−1,5
ELOVL2	Elongation of very long chain fatty acids protein 2	−1,5
KCTD6	Potassium channel tetramerisation domain containing 6	−1,5
AURKA	Aurora kinase A	−1,5
KIF18A	Kinesin family member 18A	−1,5
ARHGAP11A	Rho GTPase activating protein 11A	−1,5
CDKN3	Cyclin-dependent kinase inhibitor 3	−1,5
PRR11	Proline rich 11	−1,5
C13orf3	Chromosome 13 open reading frame 3	−1,5
SPAG5	Sperm associated antigen 5	−1,6
CA8	Carbonic anhydrase VIII	−1,6
PLK1	Polo-like kinase 1 (Drosophila)	−1,6
SGOL1	Shugoshin-like 1 (S, pombe)	−1,6
CCNA2	Cyclin A2	−1,6
CDC20	Cell division cycle 20	−1,6
NUF2	NUF2, NDC80 kinetochore complex component	−1,6
KIF20A	Kinesin family member 20A	−1,6
ANP32E	Acidic (leucine-rich) nuclear phosphoprotein 32 family, member E	−1,7
RPL22	Ribosomal protein L22	−1,7
PBK	PDZ binding kinase	−1,7
PTGES	Prostaglandin E synthase	−1,7
HEY2	Hairy/enhancer-of-split related with YRPW motif 2	−1,8
SNORD27	Small nucleolar RNA, C/D box 27	−1,8
SLC47A1	Solute carrier family 47, member 1	−1,8
ESCO2	Establishment of sister chromatid cohesion N-acetyltransferase 2	−1,9
C14orf129	Chromosome 14 open reading frame 129	−1,9
FLNA	Filamin A, alpha (actin binding protein 280)	−1,9
CTSD	Cathepsin D	−2,0
RBM24	RNA binding motif protein 24	−2,0
POLE3	Polymerase (DNA directed), epsilon 3 (p17 subunit)	−2,1
LOC440093	Histone H3-like	−2,2
AHNAK2	AHNAK nucleoprotein 2	−2,2
AREG	Amphiregulin	−2,3
RCN2	Reticulocalbin 2, EF-hand calcium binding domain	−2,3

**Table 2:** The 14 functional terms found in the cancer category of the IPA biological function analysis of 208 genes regulated by 17β-HSD1 and/or estradiol in T47D cells.

Number	Functional term
1	Cancer
2	Breast cancer
3	Delay in tumorigenesis
4	Growth of tumor
5	Incidence of tumor
6	Mammary tumor
7	Neoplasia of tumor cell lines
8	Triple-negative breast cancer
9	Tumorigenesis of breast cancer cell lines
10	Tumorigenesis of cells
11	Tumorigenesis of mammary adenocarcinoma
12	Tumorigenesis of mammary gland
13	Tumorigenesis of mammary tumor
14	Tumorigenesis of tumor cell lines

**Table 3:** The 14 functional terms found in the cell death and survival category of the IPA Biological function analysis of 208 genes regulated by 17β-HSD1 and/or estradiol in T47D cells.

Number	Functional term
1	Apoptosis
2	Apoptosis of breast cancer cell lines
3	Apoptosis of breast cell lines
4	Apoptosis of mammary epithelial cells
5	Apoptosis of mammary tumor cells
6	Apoptosis of tumor cell lines
7	Cell death
8	Cell death of tumor cell lines
9	Cell survival
10	Cell viability
11	Cytotoxicity of cells
12	Cytotoxicity of cytotoxic T cells
13	Cytotoxicity of T lymphocytes
14	Necrosis

**Table 4:** IPA network analysis of 208 genes regulated by 17β-HSD1 and/or estradiol in T47D cells from the three contrasts listed in Table 5. ***Molecules in Network***: All of the molecules that compose each network are listed. ***Score***: The score is based on a *p*-value calculation, which calculates the likelihood that the Network Eligible Molecules that are part of a network are found therein by random chance alone. Mathematically, the score is simply the negative exponent of the right-tailed Fisher׳s exact test result. For example, if the score is 3, then the there is a 1 in 1000 chance that the Network Eligible Molecules found in that network appeared there just by chance. In other words, the score is simply a measure of the number of Network Eligible Molecules in a network, and the greater the number of Network Eligible Molecules in a network, the higher the score (lower the p-value) will be. ***Focus Molecules***: This column simply indicates the number of Network Eligible Molecules per network. Since the maximum number of molecules per network is currently limited to 35, the number of Network Eligible Molecules per network cannot exceed 35. ***Top Functions***: Only the three most significant functions for each network are listed.

ID	Molecules in network	Score	Focus molecules	Top functions
1	2׳ 5׳ oas, Akt, DDX58, DDX60, DHX58, FBXO32, Fcer1, HERC5, IFIT1, IFIT3, IFITM1, IFITM3, Ifn, IFN Beta, Interferon alpha, IRF, MX1, N-cor, OAS1, OAS2, OAS3, Oas, PARP9, PBK, PI3K (family), RARRES3, RCN2, RSAD2, SEMA6A, STAT2, STAT-1/2, Thioredoxin reductase, TXNRD1, USP18, VTCN1	42	23	Antimicrobial response, inflammatory response, inflammatory disease
2	Alpha catenin, Alpha tubulin, AREG/AREGB, Cadherin, CDH10, Cg, CITED2, EPAS1, ERBB2, ERBB4, estrogen receptor, FKBP4, GNMT, GREB1, Hdac, Histone h3, Hsp70, Hsp90, ID1, MCM10, PFKFB3, PGR, Pkc(s), POLE3, PTGES, RBBP8, RNA polymerase II, RPL22, SMARCA1, SPINK4, STC2, STEAP2, TCF, TM4SF1, Ubiquitin	38	22	Connective tissue development and function, embryonic development, organ development
3	androstenediol, ARL4D, C5AR2, CD97, CSAD, CYP1A1, EID3, ESR1, FLRT3, HLA-C, ICAM3, IL6, KCNF1, KCNH1, KCTD6, L3MBTL3, LRRK2, mir-19, miR-149-3p (and other miRNAs w/seed GGGAGGG), miR-183-5p (miRNAs w/seed AUGGCAC), miR-19b-3p (and other miRNAs w/seed GUGCAAA), MTERFD3, NAALADL2, NMU, RAB27B, RAPGEFL1, RCN2, RNF135, SLC46A3, SLC47A1, Slco1a1, SOX13, SPC25, THSD7A, TMEM116	38	21	Organismal injury and abnormalities, reproductive system development and function, reproductive system disease
4	ADRB, APC (complex), AURKA, BRCA2, BUB1, calpain, CASP4, caspase, CCNA2, CCNB2, Cdc2, CDC20, Cdk, CKS2, Cyclin A, Cyclin B, Cyclin D, Cyclin E, E2f, FLNA, Ifn gamma, MAP2K1/2, NDC80, NFkB (complex), NFYB, NUF2, PLK1, PP2A, PTTG1, RAD51, Rb, RWDD2A, SGOL1, SPAG5, XAF1	33	19	Cell cycle, cellular assembly and organization, dna replication, recombination, and repair
5	AKAP8L, ANP32E, APEH, ARHGAP11A, BTN3A1, CEP152, DTX3L, FAM115A, FBXO38, FDPS, FTSJ3, HMG CoA synthase, IFRD2, INSIG2, KLF8, LGALS7/LGALS7B, LRRC41, MDGA2, NBEA, OAS3, PABPC4, PARP9, PHF7, PPM1G, PRR11, PXMP4, RBM24, RHOBTB1, RNASEH2B, RNF43, SKA3, SNRPA, SPC24, UBC, ZNF622	30	18	Hereditary disorder, neurological disease, psychological disorders
6	20s proteasome, B2M, CD8, ERAP1, ERK1/2, H-2db, HLA Class I, HLA-A, Hla-abc, HLA-B27, HLA-B, HLA-C, HLA-E, HLA-F, HLA-G, IFI27, IFI35, IFI44, IFIH1, IFIT2, IFN alpha/beta, IFN type 1, Interferon-Î± Induced, ISGF3, KIR, LGALS3BP, MHC, MHC Class I (complex), MHC CLASS I (family), MHC I-Î±, PSMB8, PSMB9, Stat1-Stat2, TAP1, Tap	28	17	Endocrine system disorders, gastrointestinal disease, immunological disease
7	AHNAK2, ANKRD27, ARL8B, BRCA2, BTN3A3, CA8, CBX8, CEP170, CKAP2L, CPAMD8, CTSL1, DDX6L, DLG5, DNPEP, ESCO2, EXOC1, FAM72A, GOLGA4, HCST, HIC1, KIF18A, KIF20A, KIF4A, LIPH, LPXN, MICB, PSMD14, RAB6A, RAB6B, SNX24, TAZ, TUBGCP2, UBC, ZNF175, ZRANB2	28	17	Connective tissue disorders, dermatological diseases and conditions, developmental disorder
8	26s Proteasome, Actin, ARAP2, BTN3A2, C8orf44-SGK3/SGK3, FGF12, GNB1, GNG11, GSK3B, GSKIP, HCP5, IFI35, IFNG, IFNL3, IL19, IRF, IRGM, LAMP3, miR-21-5p (and other miRNAs w/seed AGCUUAU), MOV10, MTORC2, Oas, PLA2, PSMB9, PSME2, RANBP3L, RSAD2, RTP4, SAMD9, SLC15A3, SLC9A3, SOCS, STAT, uric acid, USP18	26	16	Dermatological diseases and conditions, infectious disease, cell-to-cell signaling and interaction
9	AKAP6, ALCAM, APOL1, CDKN3, CREBZF, CTSD, DDIT4, FBP1, FSH, Gsk3, hemoglobin, HISTONE, Histone h4, IKK (complex), Insulin, KCNMA1, Lh, Mapk, MYC, Notch, OASL, p85 (pik3r), Pka, PRKAC, Rac, Ras, Ras homolog, ROBO2, SELENBP1, Shc, SOX2, TCR, TRIP13, Vegf, ZNFX1	25	16	Cellular growth and proliferation, tissue development, cell morphology
10	Alp, Ap1, Cbp/p300, CCL5, CDCA5, Collagen type I, Collagen type IV, Collagen(s), ELOVL2, ERK, Focal adhesion kinase, HEY2, IL1R1, Integrin, JAK, KYNU, LAMC1, Laminin1, Laminin, LDL, LTBP1, Mek, MYB, p70 S6k, Pdgf (complex), PDGF BB, Pias, PXK, Smad, Smad2/3, Sos, STAT5a/b, Tgf beta, TGFB2, THBS1	18	12	Cardiovascular disease, embryonic development, organ development
11	alpha-estradiol, androstenediol, ATP12A, ATP1B1, BCAS1, beta-estradiol, CD40, Ck2, CTSO, EGFR, EGFR ligand, Egfr-Erbb2, ERBB, ganglioside GD1a, GRM4, IER2, IFI30, IFNE, Igf, LTB, mir-146, miR-29b-3p (and other miRNAs w/seed AGCACCA), Mmp, OLFM1, PTGDS, PVRL4, RAC1, RERG, SEPP1, SERPINA6, TAP1, TP53INP1, UBP1, VGLL1, Wap	18	12	Cell morphology, cellular assembly and organization, cellular development
12	AMPK, BCR (complex), CD3, cytochrome C, F Actin, HERC6, Hsp27, Ige, IgG1, IgG, Igm, Ikk (family), IL1, IL12 (complex), IL12 (family), Immunoglobulin, Jnk, MECOM, MHC Class II (complex), MYBL1, P38 MAPK, PARP14, PARP, PI3K (complex), PLC gamma, PLSCR1, Pro-inflammatory Cytokine, Rsk, Rxr, S100A8, SRC (family), STAT1, Tlr, Tnf (family), TNFSF10	10	8	Cellular development, hematological system development and function, hematopoiesis

**Table 5:** Summary of the cell experiments and microarray analyses.

**T47D cell transfections and treatment**					
Time	Experiments	Well 1	Well 2	Well 3	Well 4
Day 1	Transfection with negative control (NC) or 17β-HSD1 (si17B1) siRNAs	NC	si17B1	NC	si17B1
Day 3	Addition of estradiol (+E2) or the *vehicle control (−)*	–	–	+E2	+E2
Day 5	Cell wash and total RNA extraction	NC	si17B1	NC+E2	si17B1+E2

**Microarray analyses**					
Three contrasts		Contrast 1: NC *vs.* si17B1	Contrast 2: NC *vs.* NC+E2	Contrast 3: NC+E2 *vs.* si17B1+E2	
Aim		List T47D genes impacted by 17β-HSD1 knockdown in steroid-deprived medium	List genes responsive to estrogen in T47D	To detect if E2 impacts gene regulation by 17β-HSD1 knockdown	
